# Near-infrared autofluorescence induced by intraplaque hemorrhage and heme degradation as marker for high-risk atherosclerotic plaques

**DOI:** 10.1038/s41467-017-00138-x

**Published:** 2017-07-13

**Authors:** Nay Min Htun, Yung Chih Chen, Bock Lim, Tara Schiller, Ghassan J. Maghzal, Alex L. Huang, Kirstin D. Elgass, Jennifer Rivera, Hans G. Schneider, Bayden R. Wood, Roland Stocker, Karlheinz Peter

**Affiliations:** 1 Baker IDI Heart & Diabetes Institute, Melbourne, VIC Australia; 2 Department of Medicine, Monash University, Melbourne, VIC Australia; 3 The Alfred Hospital, Melbourne, VIC Australia; 4 International Institute for Nanocomposites Manufacturing, University of Warwick, Coventry, UK; 5 Victor Chang Cardiac Research Institute, Sydney, NSW Australia; 6 Hudson Institute of Medical Research, Melbourne, VIC Australia; 7 Centre for Biospectroscopy, School of Chemistry, Monash University, Melbourne, VIC Australia; 8 School of Medical Sciences, University of New South Wales, Sydney, NSW Australia

## Abstract

Atherosclerosis is a major cause of mortality and morbidity, which is mainly driven by complications such as myocardial infarction and stroke. These complications are caused by thrombotic arterial occlusion localized at the site of high-risk atherosclerotic plaques, of which early detection and therapeutic stabilization are urgently needed. Here we show that near-infrared autofluorescence is associated with the presence of intraplaque hemorrhage and heme degradation products, particularly bilirubin by using our recently created mouse model, which uniquely reflects plaque instability as seen in humans, and human carotid endarterectomy samples. Fluorescence emission computed tomography detecting near-infrared autofluorescence allows in vivo monitoring of intraplaque hemorrhage, establishing a preclinical technology to assess and monitor plaque instability and thereby test potential plaque-stabilizing drugs. We suggest that near-infrared autofluorescence imaging is a novel technology that allows identification of atherosclerotic plaques with intraplaque hemorrhage and ultimately holds promise for detection of high-risk plaques in patients.

## Abstract (web summary)

Atherosclerosis diagnosis relies primarily on imaging and early detection of high-risk atherosclerotic plaques is important for risk stratification of patients and stabilization therapies. Here Htun et al. demonstrate that vulnerable atherosclerotic plaques generate near-infrared autofluorescence that can be detected via emission computed tomography.

## Introduction

Although atherosclerosis is usually described as a single disease entity with significant morbidity and mortality worldwide, not all atherosclerotic lesions or plaques are the same with vast differences in their composition and natural history^1–5^. Some plaques remain quiescent and stable for years and others become unstable and vulnerable, ultimately leading to plaque rupture. Plaque rupture is the main cause of thrombosis and occlusion of arteries leading to potentially catastrophic outcomes such as stroke and myocardial infarction (MI), the latter being the most frequent single reason of death^6, 7^. The development of a method to reliably identify high-risk atherosclerotic plaques is seen as one of the major quests of contemporary cardiovascular medicine. It would provide the opportunity to apply prophylactic treatment, either pharmacological or interventional, and thus to prevent e.g. MI and its associated substantial morbidity and mortality.

Significant efforts have been made to characterize and risk stratify carotid and coronary atherosclerotic plaques using various imaging technologies. We here propose the use of near-infrared autofluorescence (NIRAF) for the identification of high-risk atherosclerotic plaques. Investigators previously used low-wavelength (300–400 nm) fluorescence technology to assess plaque composition, detecting fluorescence signals of collagen, elastin, and oxidized low density lipoprotein, typically as components of the lipid core^8–13^. However, this technology was limited by poor specificity, as low wavelength fluorescence is not exclusively emitted by lipid core associated materials, as well as poor signal-to-noise ratio and weak tissue penetration caused by hindering tissue autofluorescence and strong photon absorption^14, 15^. In contrast, our study demonstrates that autofluorescence in the NIR range uniquely characterizes atherosclerotic plaques with intraplaque hemorrhage and may allow classification of atherosclerotic plaques into lesions of low or high risk for future cardiovascular events.

We have previously developed a mouse model that exhibits unstable plaques as seen in humans^16^. This mouse model was instrumental for the description of NIRAF as a potential indicator of an atherosclerotic plaque’s risk in causing future cardiovascular events. In addition to human CEA samples, this model allowed us to identify intraplaque hemorrhage and heme degradation products as sources of the detected NIRAF signal. Besides establishing fluorescence emission tomography (FLECT) as a preclinical tool for the detection of plaques with intraplaque hemorrhage and its potential use in testing and monitoring of plaque-stabilizing drugs, our report has the potential to support technical developments for imaging technologies that will ultimately allow to risk stratify atherosclerotic plaques in patients and, in particular, to identify plaques that are prone to cause cardiovascular events.

## Results

### NIRAF in atherosclerotic plaques

Both human and murine atherosclerotic samples were used in this study. With informed consent, human CEA specimens were collected from patients who presented to the Alfred Hospital, Melbourne, Australia with clinical indications for CEA.

**Fig. 1:**
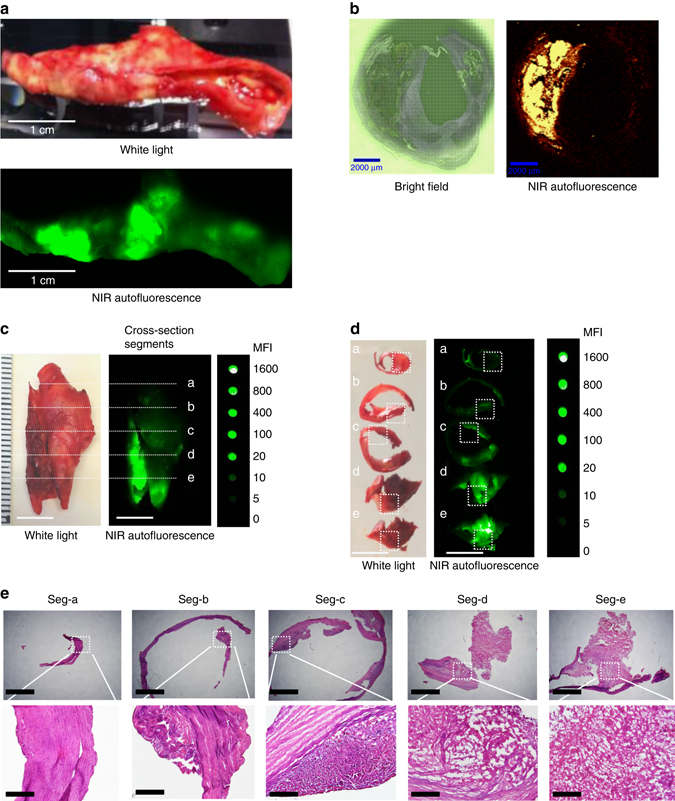
NIRAF imaging of human CEA samples and representative histology in five cross section segments. **a** Macroscopic sample of a fresh human CEA specimen under white light (*upper photo*) and fluorescence imaging using the Odyssey Infrared Imaging System (*lower image*) showing localized areas of NIRAF (*n* = 31). **b** Paraffin section of human CEA specimen (5 µm sections): Bright field (*left*) and areas of NIRAF on the WITek Raman 300R system at 785 nm excitation (*right*, *n* = 5). **c** Fresh human CEA specimen under white light (*left*) and its NIRAF image using Odyssey Infrared Imaging System (*middle*) shown in comparison with a serial dilution of IRDye800CW (Em = 794 nm) (*right*). The same sample was sectioned into five segments (seg-a–seg-e) along the *dash lines* labeled “a”–“e” as shown in **c**. Scale bars indicate 1 cm (*n* = 8). **d** These five plaque segments (seg-a–seg-e) under white light (*left*) and their corresponding NIRAF images using Odyssey Infrared Imaging System (*middle*) were again shown in comparison with the IRDye800CW (*right*). Seg-a has MFI of 2, seg-b = 10, seg-c = 20, seg-d = 200, and seg-e = 1000, respectively. Scale bars indicate 1 cm (*n* = 8). **e** Histology of these five plaque segments with increasingly advanced pathology stained with hematoxylin and eosin. Seg-a: intimal thickening only. Seg-b: some lipid accumulation. Seg-c: atheroma with multiple foam cells. Seg-d and seg-e: fibroatheroma with intraplaque hemorrhage. Scale bars indicate 500 µm in the upper row and 100 µm in the lower row (*n* = 8). MFI = mean fluorescence intensity. *Square dash box* indicates the magnification position and reference points in the original NIRAF and under white light images

To investigate human atherosclerotic plaques, 50 CEA samples freshly taken from patients in the operating theater were immediately scanned using the Odyssey Imaging System before being snap-frozen and stored at −80 °C prior to further analysis. Of which, 26 samples were from patients who presented with symptoms such as stroke or transient ischemic attacks, while 24 samples were from asymptomatic patients. Patients with symptoms can be assumed to harbor more complicated plaques, whereas asymptomatic patients underwent endarterectomy because they had high-grade, severely stenotic lesions (>70–80% luminal stenosis on vascular ultrasound or CT angiogram). Hence the CEA samples obtained will exhibit a mixture of advanced atherosclerosis including histological characteristics of unstable as well as stable plaque pathology. Thirty-one out of 50 CEA samples collected (62%) showed significant intrinsic autofluorescence (mean fluorescence intensity (MFI)>20) in the NIR range (NIRAF, Fig. 1a, c, d). To provide a comparison and scale for the MFI measurements, samples were scanned together with a serial dilution of IRDye800CW. Overall, MFI ± standard error of the mean (SEM) was 404.7 ± 83.2 for fluorescent plaques in contrast to 10.3 ± 3.2 in non-fluorescent plaques (background fluorescence) (*p* < 0.0001, unpaired Student’s *t*-test).

To confirm the fluorescence nature of the CEA specimens, an additional technology capable of NIR fluorescence detection was used. The WITec Raman 300R system (WITec, Germany) utilized an excitation laser source of 785 nm to detect autofluorescence emission signals. This was achieved by integrating the area under the curve between 4000 and 150 cm^−1^, which is equivalent to integrating the area between 794 and 1144 nm, when taking into consideration the Stokes shift using 785 nm excitation. At 785 nm no Raman bands were observed as the background fluorescence dominated the spectral profile. Hence, the images produced are based on sample autofluorescence and not Raman scattering. Both paraffin-embedded tissue sections and cryosections of CEA samples of 5 µm thickness were scanned and showed no method-dependent differences. This imaging technique was able to reproduce the findings of the Odyssey Imaging System and demonstrated the intrinsic fluorescence in CEA samples at 785 nm excitation (Fig. 1b). To further justify the chosen cut-off value of MFI>20, a detailed histological assessment of five cross section segments was provided (seg-a–seg-e, 5 mm distance between each segment, Fig. 1c and d). Seg-a showed pathological intimal thickening (MFI = 2). Seg-b revealed mild lipid accumulation (MFI = 10). Seg-c showed an atheroma with multiple foam cell macrophages (MFI = 20), while seg-d (MFI = 200) and seg-e (MFI = 1000) showed fibroatheromas with intraplaque hemorrhages (Fig. 1e). Overall, these histological features support our rationale in choosing MFI>20 as the cut-off point to discriminate plaques with morphological features of plaque instability from those without.

Murine atherosclerotic samples were obtained using the “tandem stenosis model” (TS model) of atherosclerosis in mice as described by Chen et al.^16^. This murine model is unique in its reflection of histological plaque instability as seen in humans. Briefly, a surgical ligation procedure (tandem ligation of the carotid artery, Supplementary Fig. 1) was performed to create a TS of the carotid artery of ApoE^−/−^ mice fed with high fat diet (6 weeks on western diet at 12 weeks of age). Seven weeks postoperatively, disruption of fibrous caps, intraplaque hemorrhage, intraluminal thrombosis, neovascularization, and other characteristics of plaque instability/rupture as described in humans were seen in these mice^16^ (segment I in Supplementary Fig. 1). This model was ideal for our study since it provided comparative tissue samples of unstable plaques with plaque rupture/intraplaque hemorrhage (segment I), stable plaques (segment III or V) and normal arterial wall (segment IV) in the same mouse (Supplementary Fig. 1), thereby allowing a direct comparison of plaque characteristics to each other^16^.

**Fig. 2:**
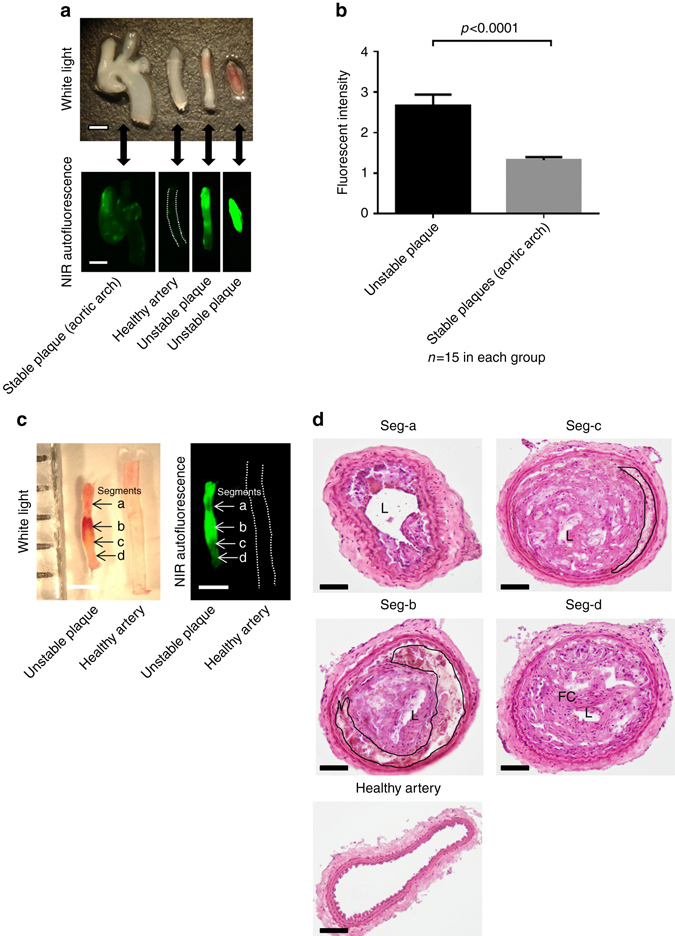
NIRAF imaging and intraplaque hemorrhage in the TS mouse model. **a** Macroscopic samples (*upper photo*) of different arterial segments from the murine TS model: from left to right: aortic arch with adjacent vessels representing stable plaques, healthy carotid artery, unstable atherosclerotic plaque with modest intraplaque hemorrhage and unstable atherosclerotic plaque with extensive intraplaque hemorrhage (both segment I of different mice) and representative NIRAF pictures (*lower images*) of their corresponding arterial segments obtained with the Odyssey Infrared Imaging System (*n* = 15). **b** Bar graph comparing NIRAF intensity (mean ± SEM) of unstable plaques to stable plaques in the tandem stenosis model in 15 mice. There was a significantly higher NIRAF signal in unstable plaques compared to stable plaques (*p* < 0.0001, Student’s *t*-test). Unstable and stable plaques were defined by histological features as outlined by Chen et al.^16^ **c** White light (*left*) and NIR images (*right*) of an unstable atherosclerotic plaque in comparison to a healthy artery, which served as background control signal. NIRAF signals were mainly co-localized with the regions of gross intraplaque hemorrhage (arrow labeled with **b**). **d** Histological images using hematoxylin and eosin staining of serial cross sections of the same samples shown in **c** (atherosclerotic plaque and normal healthy artery). Seg-a shows an intermediate lesion while seg-b exhibits extensive intraplaque hemorrhage (*black line* indicates the hemorrhage region). Seg-c also exhibits modest intraplaque hemorrhage and seg-d shows a thick cap fibroatheroma. L, lumen, FC, fibrous cap, TS, tandem stenosis. Scale bars indicate 1 mm in **a** and **c** and 100 µm in **d**

Seven weeks after TS surgery, mice were euthanized and the aortic arch, along with its main branches, harvested as previously described^16^, and scanned freshly with the Odyssey Imaging System (Fig. 2a). Intraplaque hemorrhage was a striking feature of the atherosclerotic plaques in segment I, as confirmed macroscopically as well as by hemoglobin and glycophorin A staining (Supplementary Figs. 1 and 2). Plaque segments with intraplaque hemorrhage developed clearly detectable NIRAF properties (MFI ± SEM of 2.66 ± 0.28, *n* = 15) in contrast to histologically stable plaques, which showed only minimal fluorescence (MFI ± SEM of 1.32 ± 0.08, *n* = 15) (Fig. 2b). The healthy artery served as a control (Fig. 2c). Serial cross-sections (seg-a–seg-d, approximately 750 µm distance between each segment) and histology demonstrated that seg-a represents an intermediate lesion and shows low NIRAF, seg-b an advanced lesion with extensive intraplaque hemorrhage and high autofluorescence, seg-c a fibroatheroma with intraplaque hemorrhage but to a lesser extent, and seg-d represented a thick cap fibroatheroma with low autofluorescence (Fig. 2d). These results indicate that NIRAF is associated with intraplaque hemorrhage and histological plaque instability in the TS mouse model.

### Raman spectroscopy indicates intraplaque hemorrhage as NIRAF source

**Fig. 3:**
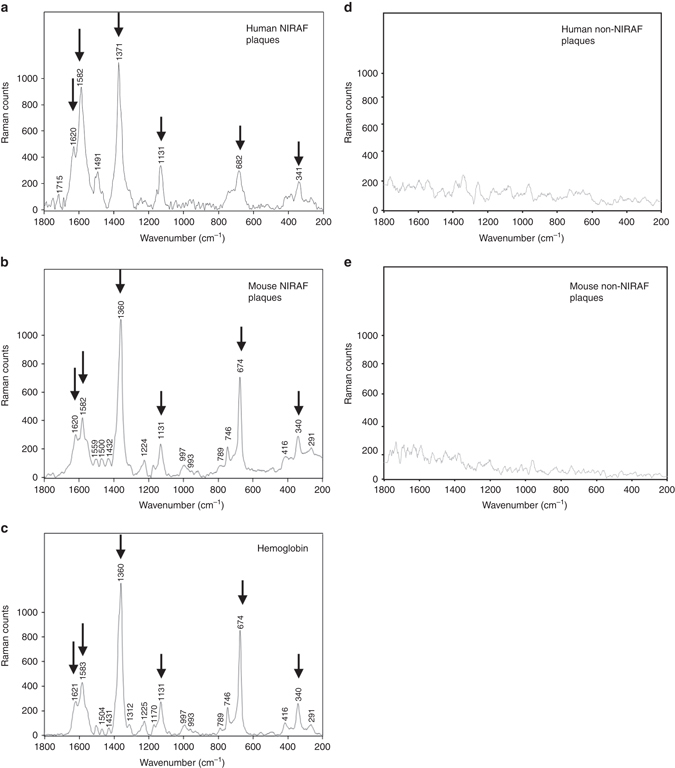
Raman signals obtained from human and murine carotid plaques with or without NIRAF. **a** Raman signals from a NIRAF human CEA sample (5 µm cryosection) showing the oxidation state marker band *ν*_4_ at 1371 cm^−1^ assigned to a symmetric pyrrole half-ring stretching vibration consistent with the Fe ion being in the ferric state associated with oxygenated hemoglobin. **b** Raman spectra from NIRAF murine carotid sample (40 µm cryosection). The spectra of the plaques appear to show a mixture of heme products as evidenced by the position of *ν*_4_, which appears at 1360 cm^−1^ indicative of ferrous heme but also shows a shoulder feature at ~1371 cm^−1^, which is associated with ferric heme indicating a mixture of oxygenated and deoxygenated hemoglobin. In oxygenated hemoglobin, which is in the ferric low spin state, this band normally appears between 1376 and 1370 cm^−1^. In deoxygenated hemoglobin, where the Fe ion is in the ferrous high spin state, this band normally appears between 1360 and 1350 cm^−1^. A number of other strong heme bands are observed in the spectra of the plaques. These include bands at 1620 cm^−1^, 1582 cm^−1^, 1544 cm^−1^ assigned to C=C and C–C bonds of the porphyrin backbone **a**, **b**. **c** Raman signals obtained from hemoglobin demonstrated almost identical peaks with murine NIRAF plaques at 1620, 1582, 1360, 1311, 1225, 789, 674, 416, 340, and 291 cm^−1^. **d** Raman signals from a human non-fluorescent plaque and **e** Murine non-fluorescent plaques demonstrated no specific chemical finger prints. Arrows indicate the major heme peaks

Once we had established NIRAF to be associated with high-risk atherosclerotic plaques, we searched for the source of NIRAF using chemical fingerprinting; comparing Raman signals obtained from fluorescent atheromatous plaques to those from non-fluorescent plaques. To record resonance Raman spectra, we had to choose an excitation wavelength well apart from the NIR fluorescence and hence we applied 413 nm. Resonance Raman spectra were recorded using a Spectra Physics Stabilite 2017 argon ion laser system coupled to a Renishaw Raman 2000 spectrometer and interfaced to a Leica Raman microscope. Resonance Raman spectra from the fluorescent atheromatous plaques showed distinct peaks (Fig. 3a and b) that closely resembled the spectrum of deoxygenated hemoglobin (Fig. 3c). We therefore assigned these peaks to heme modes. Importantly, these peaks were not detected in the plaques without NIRAF (Fig. 3d and e). Moreover, mouse plaques exhibiting NIRAF had almost identical characteristic fingerprints—except for differences in intensity (Fig. 3b and c). These findings were consistent for both human and murine unstable plaque samples. The detailed peak assignments are shown in Supplementary Table 1 including spectra for comparison from oxygenated hemoglobin, deoxygenated hemoglobin with hemin (FePPIX-Cl), and hematin (FePPIX-OH)^17^, recorded at the same excitation wavelength. The spectra obtained from the fluorescent plaques in mice and humans reveal a mixture of heme products as evidenced by the position of *ν*_4_, which appears between 1360 and 1371 cm^−1^ and is assigned to a symmetric pyrrole half-ring stretching vibration^17^. This band is known as the oxidation state marker band because it is sensitive to the oxidation state of iron^17^. In oxygenated hemoglobin, which is in the ferric low spin state, this band normally appears between 1376 and 1370 cm^−1^. In deoxygenated hemoglobin, where the Fe ion is in the ferrous high spin state, this band normally appears between 1360 and 1350 cm^−1^. Figure 3c shows the spectrum of hemoglobin recorded using 413 nm excitation, which shows a strong band at 1360 cm^−1^ and a shoulder at 1372 cm^−1^, indicating this contains a mixture of ferrous and ferric iron. The appearance of *ν*_4_ between 1360 and 1371 cm^−1^ in the fluorescent plaques indicates that the heme-based compounds in these plaques are also likely to be a mixture of oxidation states as this lies between the wave number values expected for ferric and ferrous heme. The likely origin of these mixed redox states of heme in atheromatous plaques is the oxidation and reduction of hemoglobin at the sites of intraplaque hemorrhage. A number of other strong heme bands are also observed in the spectra obtained from the plaques. These include bands at 1620 cm^−1^, 1582 cm^−1^, 1544 cm^−1^ assigned to C=C and C–C bonds of the porphyrin backbone and other bands at 753 and 666 cm^−1^ assigned to pyrrole breathing and deformation modes, respectively^17^. Due to the inherent nature of atherosclerosis that contains a mixture of different biological tissues, the Raman signals of heme degradation products from the plaques are likely to be shifted to some extent by co-existing compounds within the plaque rather than exhibiting classical Raman spectra of pure chemicals.

In the event of intraplaque hemorrhage, inflammatory cells e.g. macrophages phagocytose extravasated red blood cells (RBCs), leading to induction of heme oxygenase-1 (HO-1). Heme derived from the ingested RBC hemoglobin is then metabolized to biliverdin via the enzyme HO-1. Biliverdin is subsequently reduced to bilirubin via biliverdin reductase. In order to investigate this further, we obtained pure standards of hemoglobin, protoporphyrin IX, biliverdin and bilirubin to cover the whole spectrum of heme metabolism) and determined their Raman spectra. The spectra of biliverdin and bilirubin differed markedly from those of hemoglobin and protoporphyrin IX because the porphyrin structure is no longer present in biliverdin and bilirubin. Overall, Raman spectra of atherosclerotic plaques with NIRAF are clearly dominated by hemoglobin signals (Fig. 3, Supplementary Fig. 3).

### NIRAF of heme precursors and heme degradation products

Based on the resonance Raman spectra obtained from atheromatous plaques, intraplaque hemorrhage is considered to be the likely source of the autofluorescence signals. Hence fluorescence characteristics at the NIR range of blood and its related products (fresh blood, old blood, oxidized RBCs, hemoglobin and its degradation products to reflect the stages of biological degradation process, when hemorrhage occurs inside the plaque) were investigated using the Odyssey Imaging System.

Samples of fresh and old blood obtained from mice were also scanned using the Odyssey System. No fluorescence signals were detected in the NIR range, indicating blood by itself, either fresh or old, is not responsible for the NIRAF found in atherosclerotic plaques (Supplementary Material, Supplementary Fig. 4).

**Fig. 4:**
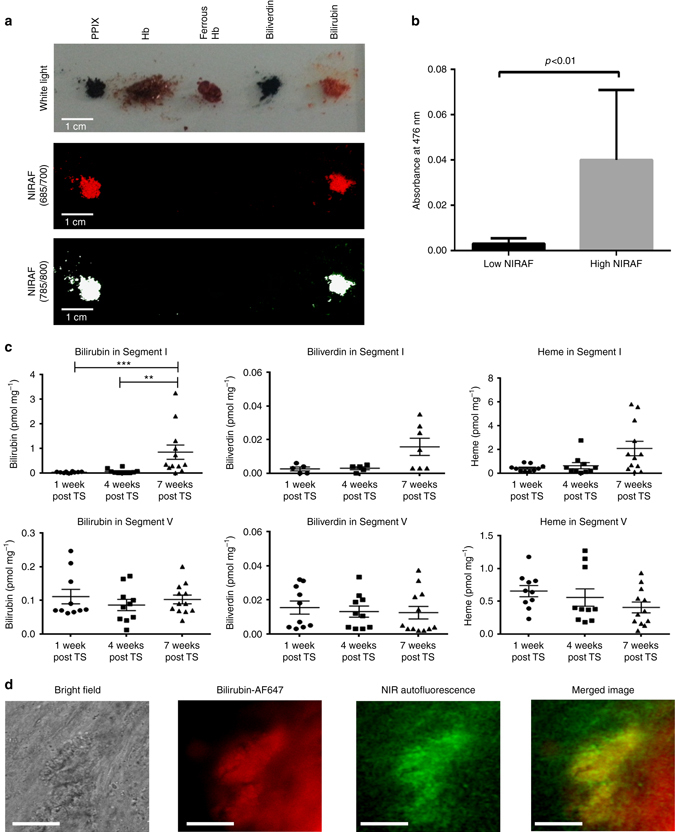
Bilirubin as potential source of NIRAF. **a** NIRAF image of tetrapyrrole-containing compounds involved in heme metabolism using the Odyssey Infrared Imaging System. NIRAF signals in both channels (Ex 685 nm, Em >700 nm; Ex 785 nm, Em >800 nm) were detected from protoporphyrin IX and bilirubin, while hemoglobin, ferrous stabilized hemoglobin and biliverdin did not show any significant NIRAF. **b** Bar graph of mean absorbance ± SEM suggestive of the differences in bilirubin content in the high and low NIRAF human CEA plaque segments (seven in each group). Plaque segments with high NIRAF had significantly higher bilirubin content (*p* < 0.01, unpaired Student’s *t*-test). **c** Mass spectrometry measurements of bilirubin, biliverdin and heme concentrations in unstable (TS segment I) vs. stable plaques (TS segment V) at 1, 4, and 7 weeks post TS surgery. The bilirubin concentration is significantly elevated in the unstable plaque 7 weeks post TS surgery (**p < 0.05, ***p < 0.01, Kruskal–Wallis test followed by Dunn’s multiple comparisons test. The heme and biliverdin concentrations also shows a similar trend, although statistically not significant (*p* = 0.061, *p* = 0.18, Kruskal–Wallis test followed by Dunn’s multiple comparisons test). Bilirubin, biliverdin and heme concentrations remained unchanged in the stable plaques. **d** Fluorescence microscopy suggestive of the co-localization of bilirubin and NIRAF (Ex: 740/13 nm, Em: 809/81 nm) in human unstable plaques. From left to right: bright field microscopy image, fluorescence image of anti-bilirubin antibody labeled with Alexa 647 (*red color*), NIRAF image (*green color*), merged image (overlaying bilirubin fluorescence image and NIRAF image) are shown. Bilirubin and NIRAF co-localization can be seen in the merged image (*yellow color*). Experiments were performed three times and one representative example is shown. Scale bar indicates 50 µm

NIRAF of the commercially available protoporphyrin IX, hemoglobin, ferrous-stabilized hemoglobin, biliverdin and bilirubin (5 mg each) was then explored. While hemoglobin, ferrous-stabilized hemoglobin and biliverdin did not exhibit any fluorescence on the Odyssey System, the heme precursor protoporphyrin IX and the final heme degradation product bilirubin revealed intrinsic fluorescence in the NIR range (Fig. 4a).

### Bilirubin extraction from the plaque tissues with autofluorescence

Seven human CEA plaque samples with NIRAF and seven samples of human carotid arterial segments without NIRAF were excised and weighed (120 mg each). A standard chloroform-based extraction was used to extract bilirubin from these samples^18, 19^ and bilirubin concentration (represented as absorbance at 476 nm) in the extracted fluid was measured with a Cary 60 UV–vis Spectrophotometer (Agilent Technologies). As demonstrated in Fig. 4b, the arterial segments without NIRAF showed minimal absorbance (all below 0.007 which was the cut-off value for detection of bilirubin) (mean absorbance ± SEM of 0.003 ± 0.001). The plaque samples with NIRAF showed absorbance more than 10 times higher (mean absorbance ± SEM of 0.040 ± 0.012). These findings support the hypothesis that NIRAF of plaques originates from heme degradation in areas of intraplaque hemorrhage.

### Mass spectrometry of heme and its degradation products in the TS model

Mass spectrometry offers the possibility to determine the concentrations of heme and its degradation products (biliverdin and bilirubin) in small amounts of tissues, such as plaques of the TS mouse model. The heme, biliverdin and bilirubin content in histologically defined unstable (segment I) and stable plaques (segment V)^16^, collected at 1, 4, and 7 weeks post TS surgery, was determined using liquid chromatography–tandem spectrometry (LC–MS/MS) on an Agilent 1290 binary pump connected to a 6490 triple quadrupole mass spectrometer (Agilent Technologies, Santa Clara, USA). The content of heme, biliverdin and bilirubin in stable plaques remained low and unchanged at all time points (Fig. 4c). In contrast, bilirubin content significantly increased in unstable plaques (segment I) at 7 weeks, compared to 1 week or 4 weeks post TS (Fig. 4c). Heme and biliverdin also showed an increase at 7 weeks; however this was not statistically significant (Fig. 4c). At 7 weeks post TS surgery, the mean bilirubin content in unstable plaques (segment I) was ~1 pmol mg^−1^ whereas the mean heme content was approximately 2 nmol mg^−1^. The finding of higher bilirubin contents in unstable plaques in TS mice was consistent with the bilirubin extraction data from human CEA samples.

### Bilirubin supports a broad range of NIRAF

Using the IVIS Lumina Series II Imaging System (Caliper Life Sciences) we compared the NIRAF characteristics of hemoglobin and bilirubin over a wide range of excitation wavelength. Whereas hemoglobin shows again no NIRAF, bilirubin shows NIRAF at a broad range of different excitation wavelengths (605, 640, 675, 710, and 745 nm) with detection of NIR signals above 810 nm (Supplementary Fig. 5, including bilirubin as control dissolved at various concentrations). Bilirubin’s NIRAF was further confirmed by strong fluorescence signals in the liver (with known abundance of bilirubin, Supplementary Fig. 6). Overall, bilirubin’s NIRAF characteristic is highly favorable as it provides substantial flexibility for device development.

### Co-localization of NIRAF and bilirubin

Lastly, co-localization of NIRAF signals and bilirubin in human high-risk plaques was confirmed in fluorescence microscopy (NIR filter: Ex: 740/13, Em: 809/81) after staining CEA samples with Alexa 647 dye-labeled anti-bilirubin antibody (Cy5 filter: Ex: 650/13, Em: 684/24; Fig. 4d) (Supplementary Materials).

### Evidence of intraplaque hemorrhage in NIRAF plaques

**Fig. 5:**
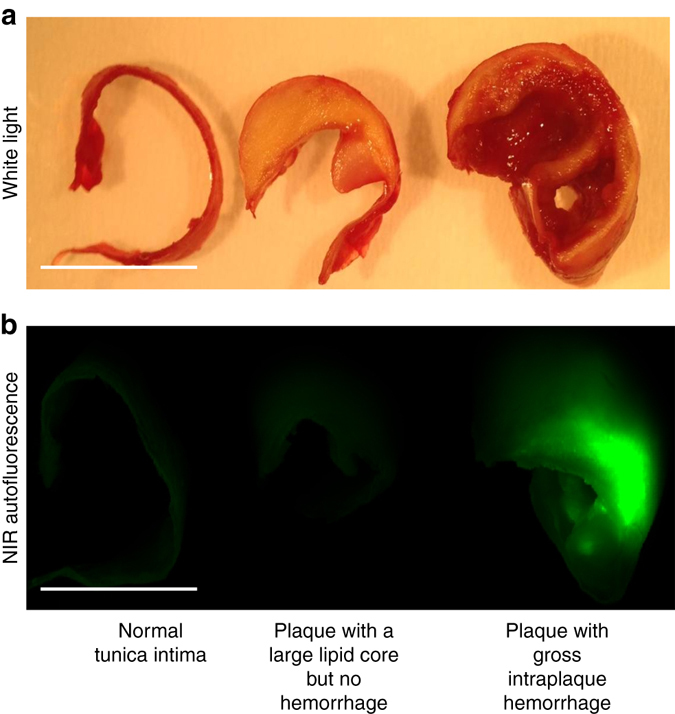
Human carotid arterial segments with their corresponding fluorescence images. **a** shows representative macroscopic sections of fresh human carotid endarterectomy samples (from left to right: normal tunica intima-the innermost layer of the arterial wall, atheromatous plaque with a large lipid core but no intraplaque hemorrhage and atheromatous plaque with intraplaque hemorrhage, respectively). **b** Corresponding images of the same tissue samples scanned on the 800 channel (Ex 785 nm, Em >800 nm) of the Odyssey Infrared Imaging System showing bright NIRAF associated with intraplaque hemorrhage within the center of the plaque. Of note, the fresh blood on the surface of the plaques does not cause NIRAF. Experiments were performed eight times and one representative example is shown. Scale bars indicate 1 cm

**Fig. 6:**
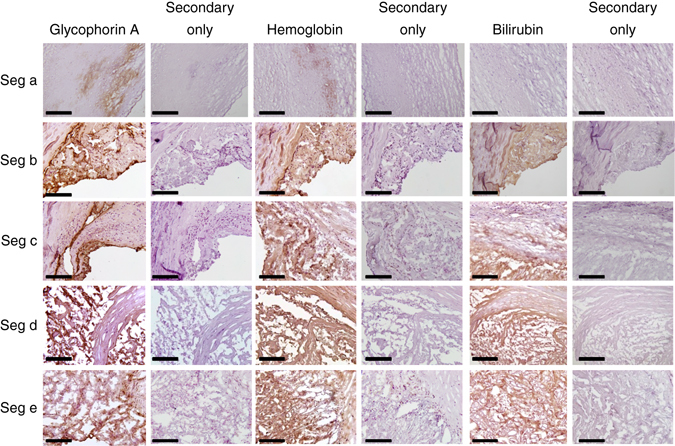
Immunohistochemical staining of intraplaque hemorrhage markers in human CEA sample in five cross-section segments (as shown in Fig. 1d and e). From left to right: Glycophorin A staining, secondary antibody only control for glycophorin A, hemoglobin staining, secondary antibody only control for hemoglobin, bilirubin staining, secondary antibody only control for bilirubin. Bilirubin was absent in seg-a but positive staining is seen in other segments, most pronounced in seg-d and seg-e. *Brown color* indicates a positive staining. Controls were performed with secondary antibody only. Experiments were performed eight times and one representative example is shown. Scale bars indicate 50 µm

Human samples of normal tunica intima as the innermost layer of the arterial wall, atheromatous plaques with a large lipid core without intraplaque hemorrhage, and atheromatous plaques with intraplaque hemorrhage were scanned using the Odyssey System. Significant NIRAF was seen only in plaques with intraplaque hemorrhage but not in the normal tunica intima or lipid-rich plaques without hemorrhage (Fig. 5a and b). Subsequently, human CEA samples with focal areas of NIRAF were sectioned into 5 different segments (seg-a–e) and NIRAF and histology of each segment obtained (Fig. 1c–e and Fig. 6). These segments with varying degrees of NIRAF intensity (seg-a shows a MFI of 2, seg-b of 10, seg-c of 20, seg-d of 200, and seg-e of 1000, respectively) were tested for immunohistochemical evidence of intraplaque hemorrhage using antibodies to glycophorin A (a major RBC membrane antigen), the hemoglobin alpha subunit, and bilirubin (Supplementary Materials). Positive staining for glycophorin A and hemoglobin was most pronounced in seg-d (with MFI of 200) and seg-e (with MFI of 1000) and minimal in seg-a (with MFI of 2), while bilirubin staining was entirely negative in seg-a and of highest intensity in seg-d and e (Fig. 6).

During the process of hemoglobin breakdown, iron will be released and is expected to be present as hemosiderin in the areas of intraplaque hemorrhage. Iron staining based on the Prussian blue reaction (using an iron stain kit) was performed to test the presence of iron/hemosiderin in human CEA specimens with or without NIRAF (see Supplementary Materials for the detailed description of iron staining). Staining was strongly positive in the CEA samples with NIRAF and only minimal in the CEA samples without NIRAF, indicating that areas of fluorescence match the areas of intraplaque hemorrhage (Supplementary Fig. 7).

### Monitoring plaque characteristics induced by pharmacological interventions

Heme oxygenase 1 expression has been demonstrated in atherosclerotic lesions in both animal and human studies and it has been implicated to be atheroprotective^20, 21^. Cheng et al.^22^ demonstrated that HO-1 induction by cobalt protoporphyrin IX (CoPPIX) impeded atherosclerotic lesion progression into vulnerable plaques in ApoE^−/−^ mice, resulting in reduced size of the necrotic cores, less intraplaque lipid accumulation and an increased ratio of fibrous cap thickness/intimal vascular smooth muscle cells, whereas inhibition of HO-1 by zinc protoporphyrin IX (ZnPPIX) augmented plaque instability. We hypothesized that the manipulation of HO-1 activity by ZnPPIX and CoPPIX will be an ideal test to evaluate whether the newly detected NIRAF characteristic would be suitable to detect and monitor changes in histological plaque instability.

**Fig. 7:**
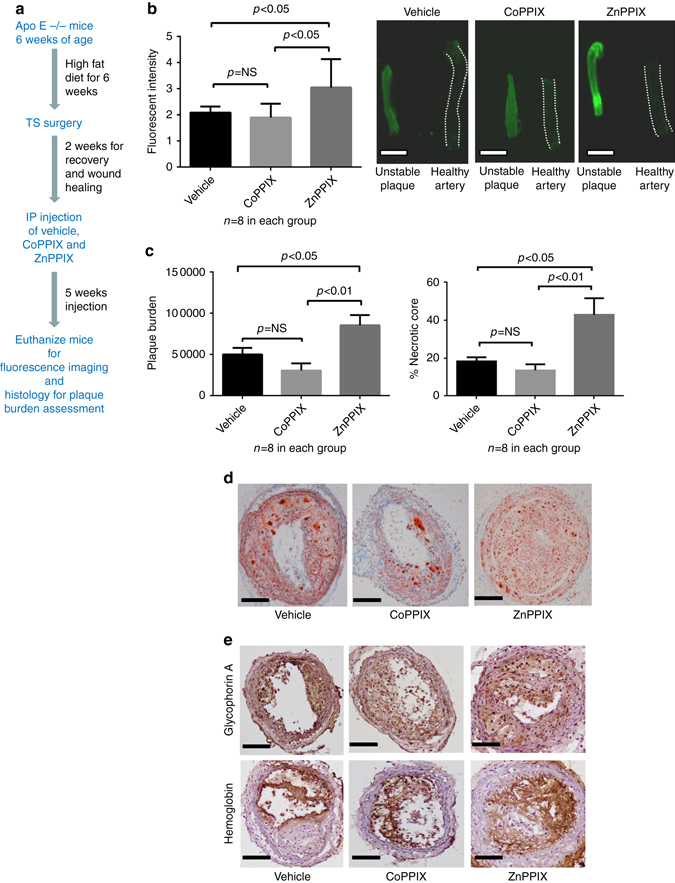
HO-1 modulation of plaque characteristics in the murine TS model. **a** The figure depicts the summary timeline of the experiment. Tandem stenosis surgery was performed on ApoE-/- mice (6 weeks on western diet at 12 weeks of age). Cobalt protoporphyrin IX (CoPPIX, HO-1 inducer) and Zinc protoporphyrin IX (ZnPPIX, HO-1 inhibitor) were used for HO-1 enzyme modulation. Two weeks after the TS surgery, intraperitoneal injections of ZnPPIX or CoPPIX or the vehicle were performed every second day for 5 weeks (*n* = 8 in each group). The mice were killed after 5 weeks of injection for analysis of NIRAF, plaque burden and necrotic core. **b** Bar graph comparing mean fluorescence intensity ± SEM of unstable plaque segments in three groups of the TS model (vehicle alone, HO-1 inhibitor ZnPPIX, HO-1 inducer CoPPIX). There was a significantly higher fluorescence signal in unstable segments of the ZnPPIX group compared to the vehicle group as well as the CoPPIX group (*p* < 0.05, Kruskal–Wallis test followed by Dunn’s multiple comparisons test). **c** Bar graph demonstrating the plaque burden (mean cubic microliter ± SEM; one way ANOVA, left graph) and necrotic core (mean % of lesion area ± SEM; Kruskal–Wallis test followed by Dunn’s multiple comparisons test, right graph) in three different groups of the TS mouse model. The plaque burden and necrotic core in the ZnPPIX group were significantly higher than the other two groups. **d** Histological sections depicting the representative pictures of three different groups stained with Oil Red O. **e** Immunohistochemistry staining of glycophorin A and hemoglobin in the unstable plaques from three groups further confirmed the presence of red blood cells and hemoglobin. Scale bar indicates 1 mm in **b** and 100 µm in **d** and **e**

Tandem stenosis surgery was performed on ApoE^−/−^ mice (6 weeks on western diet at 12 weeks of age). CoPPIX (HO-1 inducer) and ZnPPIX (HO-1 inhibitor) were used for HO-1 enzyme modulation. Two weeks after the TS surgery, intraperitoneal injections of heme oxygenase modulators (5 mg/kg of ZnPPIX or CoPPIX, dissolved in 0.2 M NaOH with PBS (without Ca^2+^/Mg^2+^) at pH 7.4) were performed as described by Cheng et al.^22^ every second day for 5 weeks. The mice were sacrificed after 5 weeks of intraperitoneal injections. The timeline of this experiment is shown in Fig. 7a. Three groups of 8 mice each were used (one group receiving ZnPPIX injection, one group receiving CoPPIX injection and one group receiving the vehicle injection; the vehicle being 0.2 M NaOH with PBS (without Ca^2+^/Mg^2+^) at pH 7.4).

The unstable plaques (segment I of the TS model) from all three groups of mice were freshly harvested and imaged using the Odyssey Imaging System. The ZnPPIX group showed significantly higher fluorescence intensity (MFI ± SEM of 3.05 ± 0.38) compared to the other two groups (MFI ± SEM of 1.90 ± 0.19 in the CoPPIX group and MFI ± SEM of 2.08 ± 0.23 in the vehicle group) (Fig. 7b). The carotid arterial segments were then embedded in “optimal cutting temperature” compound and stored at −80 °C. Quantification of the plaque burden and necrotic core was performed by Optimus 6.2 VideoPro software using Oil Red O and H&E staining, respectively. The ZnPPIX group demonstrated significantly higher plaque burden and percentage of necrotic core of total lesion, as a measure of plaque instability, compared to the other two groups (mean plaque burden in cubic microliters ± SEM of 85,300 ± 12,400 in the ZnPPIX group, 30,400 ± 800 in the CoPPIX group and 49,800 ± 7900 in the vehicle group) (Fig. 7c and d). We saw only a statistically non-significant trend in reduction of plaque burden, necrotic core and also NIRAF under the CoPPIX treatment, which may be related to this study arm being underpowered. Nevertheless, overall NIRAF measurements seem to reflect changes (or no changes) in plaque instability. Immunohistochemical staining of hemoglobin and glycophorin A in the three treatment groups further confirmed the presence of RBCs and hemoglobin in the regions of plaque instability (segment I) (Fig. 7e).

These findings indicate that changes in histologically defined plaque instability can be detected and monitored by NIRAF measurements.

### Fluorescence emission computed tomography imaging of the TS model

**Fig. 8:**
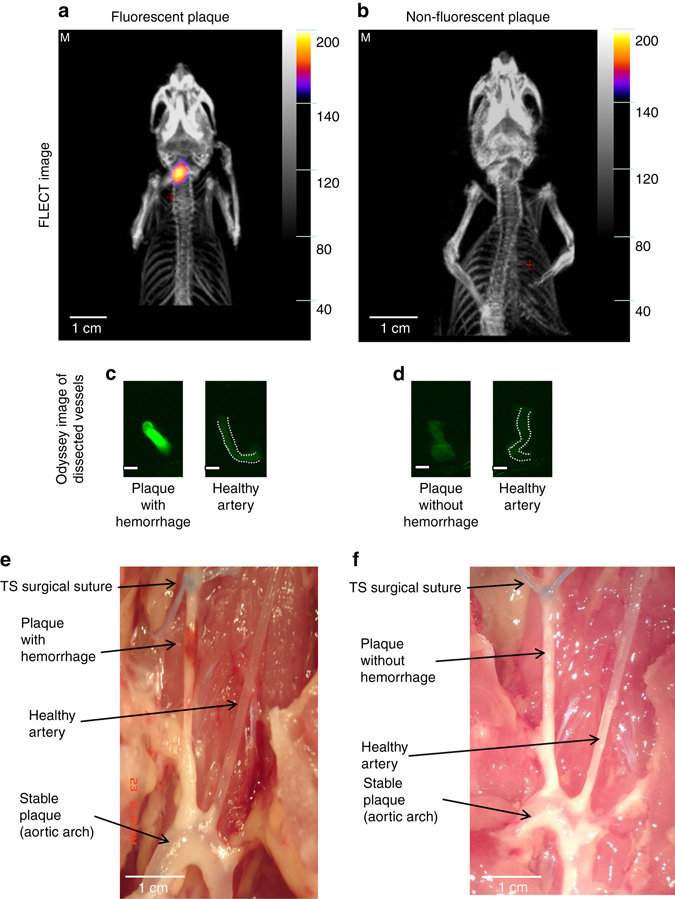
Fluorescence emission computed tomography (FLECT) and anatomical images of mice following TS ligation. **a** FLECT image of a mouse with unstable carotid artery plaque (segment I) and intraplaque hemorrhage. CT image reconstruction showed NIRAF originating from the carotid artery (segment I). **b** FLECT image of another mouse that did not develop intraplaque hemorrhage in segment I (only ~50% of mice develop intraplaque hemorrhage after TS surgery^16^) and did not exhibit significant NIRAF. **c** The dissected unstable plaque of the right carotid artery (*left*) was scanned together with the healthy left carotid vessel (*right*) using the Odyssey Infrared Imaging System. NIRAF signal was seen in the unstable plaque with hemorrhage. **d** The dissected plaque without intraplaque hemorrhage and its contralateral heathy carotid vessel were also collected and scanned. No NIRAF was detected in these vessels. **e** Anatomical depiction of a TS mouse with macroscopically visible hemorrhage. **f** Anatomical depiction of a TS mouse without hemorrhage (suture appears *blue*). Note: **a**, **c**, **e** were from a TS mouse with hemorrhagic phenotype. **b**, **d**, **f** were from a TS mouse with non-hemorrhagic phenotype. Experiments were performed 10 times and one representative example is shown. Scale bars indicate 1 mm in **c** and **d**

FLECT scanning (TriFiol, Chatsworth, CA) was used to obtain three-dimensional NIRAF images of mice with the TS model, exhibiting high-risk atherosclerotic plaques. NIRAF was detected in TS mice exhibiting intraplaque hemorrhage (Fig. 8a), but not in mice with the same TS surgery that did not develop intraplaque hemorrhage (Fig. 8b, note: ~50% of mice developed intraplaque hemorrhage in segment I of the TS model^16^). The subsequent CT images confirmed NIRAF signals are localized to segment I of the carotid artery (Fig. 8a). To further demonstrate the anatomical position of the NIRAF, we provide the three dimensional reconstruction of the FLECT (Supplementary Movies 1 and 2). Mice were then euthanized and the carotid artery segments harvested and scanned with the Odyssey System, which confirmed the presence of NIRAF (Fig. 8c and d). Representative macroscopic pictures depict the distinct phenotypes of atherosclerotic plaque (Fig. 8e) either with or without hemorrhage after TS surgery (Fig. 8f).

## Discussion

Although some of the components of plaques such as collagen, elastin, some extracellular lipids and ceroids/lipofuscin have been shown to exhibit intrinsic fluorescence when excited in the violet and blue range (325–475 nm)^8–13^, fluorescence measurements of atherosclerotic plaques in the NIR range reported here has substantial technical advantages compared to lower wavelength imaging. NIR fluorescence imaging (650–900 nm) encounters markedly less photon absorption, has better tissue penetration and less background autofluorescence of tissues^15^. Various extrinsic fluorophores for a variety of molecular targets (matrix metalloproteinases^23, 24^, macrophages^24^, and cathepsins^15, 24–27^) have been tested in animal models of atherosclerotic plaque development in mice^23, 27^ and New Zealand rabbits^25, 26^ with a focus on intravascular probes. Our study describes and characterizes NIR fluorescence signals in atheromatous plaques without the requirement of external fluorophores. The areas of NIRAF coincide with the areas of intraplaque hemorrhage with higher concentrations of bilirubin, the major breakdown product of heme. Our study indicates the source of NIRAF likely to be the heme degradation product bilirubin.

Porphyrin derivatives have long been described as fluorophores with emission spectra reaching red and infrared ranges^28–33^. Some of the NIR dyes were synthesized using porphyrin-based compounds^29^. Bilirubin was also known to exhibit intrinsic fluorescence in the 400–500 nm range^34^ and was recently also reported to have an emission peak at 660 nm while excited at 615 nm, which is consistent with our findings^35^. Naturally occurring protoporphyrin IX, a precursor of heme, showed autofluorescence in the NIR spectrum^36^. Fluorescence signals of these compounds are not constant in intensity and can change markedly with protein binding. Bilirubin’s fluorescence is enhanced with albumin binding^37–39^ or binding with metals (e.g., tetrapyrroles fluorescence quenches significantly with iron^40^ and bilirubin fluorescence quenches with gold^41^). Even interaction with human erythrocyte ghosts seems to enhance the fluorescence of bilirubin^42^. In our experiments, bilirubin displayed NIRAF on the Odyssey Infrared Imaging System. Bilirubin is sufficient for NIRAF generation, although other atheroma-based molecules, including the heme precursor protoporphyrin IX, could be additional sources of NIRAF. Hence it is likely that heme degradation products accumulate in the areas of intraplaque hemorrhage and mixed with various proteins, lipids and necrotic debris then become a source of NIRAF.

Interestingly, Raman spectra collected from plaques with NIRAF showed predominantly hemoglobin signals, rather than bilirubin signals. Most likely the Raman spectra are overloaded by hemoglobin. Mass spectrometry-based measurements of heme and bilirubin in unstable carotid plaques from segment I of TS mice (Fig. 4c) showed more than 1000-fold higher heme than bilirubin concentrations. The high concentration of heme in these plaques and its resonant enhancement at 413 nm are the likely explanations for Raman signals of fluorescent plaques to predominantly represent hemoglobin.

Intraplaque hemorrhage is one of the classical histological features of plaque instability^43–45^ and it is believed to be caused either by rupture of thin fibrous caps or by leaky vessels as a result of extensive neovascularisation of unstable atheromatous plaques^46^. Intraplaque hemorrhage has been shown not only to be a common feature of larger arteries but also of high-risk coronary plaques. Kolodgie et al.^47^ found that 77% of the high-risk, thin-cap fibroatheromas exhibit intraplaque hemorrhage. In a study of 57 patients who died of coronary artery disease, Virmani et al.^48^ found extravasated erythrocytes in coronary plaques of 84% of patients. Recent studies indicate that intraplaque hemorrhage could also be a trigger, rather than just a marker, of plaque instability, by playing a major role in plaque progression with resultant accumulation of erythrocyte membranes, cholesterol crystal generation and retention, enlargement of necrotic cores, promoting oxidant and proteolytic activities, leukocyte infiltration, and inflammation in plaques^47, 49–51^.

There are several histological features that characterise an unstable and high-risk atherosclerotic plaque: Thin fibrous cap of <65 µm (giving rise to the term thin-cap fibroatheroma (TCFA)); large necrotic core; increased plaque inflammation; positive vascular remodeling; increased neovascularization by vasa vasorum; and intraplaque hemorrhage^43, 45, 52^. Despite extensive advances in the field of cardiovascular medicine and imaging technologies, the ability for early detection of the high-risk plaques remains elusive. Several invasive plaque-imaging techniques have been developed with the aim to detect the various characteristics of histologically defined unstable plaques (such as TCFA). However, most of these currently used methods are able to detect only some, but not all, histological features of unstable plaques.

Using intravascular ultrasound with virtual histology (IVUS-VH) Stone et al.^53^ showed the major adverse cardiovascular event rate for non-culprit lesions, which were classified as TCFA, exhibiting a plaque burden of >70% and minimal luminal area of  ≤ 4 mm^2^, was 18.2% at 3.4 years follow-up. While this clearly demonstrates that the use of IVUS-VH has some value in predicting future adverse cardiovascular events, it is still far from ideal with a relatively poor predictive value, highlighting its limitations in detecting all of the histological criteria of unstable plaques.

Optical coherence tomography (OCT) with its high resolution (10–15 µm) is well-suited for detection of thin fibrous caps, macrophages and necrotic core but is not well designed for reliable detection of positive remodeling or plaque burden^43, 54–57^. Fujii et al.^58^ found that the mean value for minimum fibrous-cap thickness within TCFAs was 57.4 ± 5.4 µm in 35 MI patients, and 55.9 ± 7.3 µm in 20 stable angina patients (*p* = 0.4), indicating that by itself TCFA, detected by OCT, is probably not sufficient to differentiate low-risk from high-risk atherosclerotic plaques.

Near-infrared spectroscopy (NIRS) has been shown to be able to detect lipid core-containing plaques in both, ex vivo and in vivo studies^59–61^. Madder et al.^62^ recently published a study using NIRS to identify the composition of target lesions in patients with acute coronary syndrome (28 patients) vs. stable angina (32 patients). Nearly 50% of patients with stable angina showed lipid core plaques in this study, casting doubts on its ability to identify the plaques with high risk for cardiovascular events.

Other emerging methods including angioscopy (visualizes surface appearance of the plaque) and thermography (detects metabolic activity of the plaque) are reporting individual characteristics of atherosclerotic plaques but have not been proven to provide predictive value yet. Intravascular application of Raman spectroscopy is also technically challenging due to its low signal-to-noise ratio and poor tissue penetration^63^.

Non-invasive MRI has been used to identify intraplaque hemorrhage and vasa vasorum in the carotid arteries with a prognostic significance^64–66^. But MRI of coronary arteries has been challenging due to their small diameter, deep location in the chest and their motions with cardiac cycle and diaphragmatic movements^67^. Intravascular MRI to image vulnerable plaques, especially in the coronary arteries, is still at an early development stage although holding promise to ultimately being able to detect intraplaque hemorrhage.

NIR fluorescence imaging catheters have been tested in rabbit models of atherosclerosis without the detection of significant NIR fluorescence signals unless external fluorophores were used^25, 26^. However, it should be noted that these rabbit models and other animal models of atherosclerosis may not represent unstable atherosclerotic plaques as seen in our TS mouse model or in humans. Hence plaques in rabbit and other larger animal models may not harbor significant areas of intraplaque hemorrhage, which we see as the central prerequisite for NIRAF (Fig. 8).

Very recently, a pioneering dual-modality OCT-NIRAF (excitation 633 nm and detection between 675 and 950 nm) optical catheter system has been developed for the investigation of human coronary arteries^68^. NIRAF signals were initially described by Wang et al.^68^ ex vivo in plaques of human coronary arteries characterized by necrotic cores. Ughi et al.^69^ went on and elegantly demonstrated NIRAF focally in plaques with high-risk morphological features in a first study in humans involving intracoronary fluorescence imaging in 12 patients. Importantly, these studies support the relevance of our findings and demonstrate the feasibility and potential of intracoronary NIRAF imaging in humans. The association of NIRAF with the necrotic core potentially points to intraplaque hemorrhage as a known crucial factor in plaque progression including enlargement of necrotic cores^49^. Based on the broad spectrum of excitation as shown by us, bilirubin is a potential source of NIRAF at the excitation wavelength used by the described OCT-NIRAF device^68^. However, other sources such as lipids and lipoproteins modified by oxidative stress could also contribute to NIRAF associated with high-risk coronary plaques as discussed by Wang et al.^68^. Notably, in a recent study, Verjans et al.^70^ showed only minimal NIRAF in three human CEA plaques detected by an intravascular NIRF-OCT system, compared to five subjects who received Indocyanine Green, a targeted NIRF agent (Ex 740 nm, Em 790 nm). While the higher emission wavelength (750–800 nm range) may be well suited for targeted NIR fluorescence agents, it might be less suited for detection of NIRAF, such as successfully reported at a range between 633 and 700 nm^68, 69^. Further in vivo intracoronary imaging studies based on these findings will help to determine the clinically most suitable NIRAF detection wavelengths for the identification of high-risk atherosclerotic plaques.

While clinicians and scientists alike are pursuing to improve intravascular imaging technologies to identify high-risk plaques, there are some studies already investigating the feasibility, safety and efficacy of pre-emptive, prophylactic stenting in potentially high-risk, but non-obstructive coronary lesions^71^. However, in general implanting stents is not without risks, including death, MI, emergency bypass surgery, stroke and bleeding as well as stent thrombosis (acute or late), with a very high mortality rate. Hence for prophylactic stenting (plaque sealing) of silent, non-obstructive lesions to become a feasible treatment option, a reliable imaging technology is required that has a high predictive value for the risk of cardiovascular complications.

Although some of the currently available intravascular imaging modalities or their combination have their own merits and are able to identify some aspects of high-risk plaques (for example, lipid core in NIRS, thin cap and monocytes in OCT and plaque burden in IVUS, etc.)^72^, none of these technologies is generally accepted to reliably detect high-risk plaques. We here describe NIRAF, which characterizes high-risk plaques with intraplaque hemorrhage both in mice and humans, as such potentially highly promising technology. We also identify a potential source of NIRAF, bilirubin; being generated by heme degradation. Our study demonstrating NIRAF in the areas of intraplaque hemorrhage, which is clearly a critical element of plaque instability, provides a much-needed new foundation in the field of intravascular imaging of high-risk plaques. Fluorescence imaging in preclinical models can now be used to assess and monitor potential plaque stabilizing drugs, a technology long sought-after by the pharmaceutical industry. In humans, the feasibility of NIRAF measurements to risk stratify atherosclerotic plaques and thus to provide a basis for decision-making on preventative treatment will have to be tested in large randomized and controlled clinical trials. Such trials will then have to determine whether NIRAF quantification holds up to the promise of ultimately making a difference in cardiovascular mortality and morbidity.

In our study, a cut-off in MFI was used to categorize whether or not human CEA samples had significant NIRAF. Although this cut-off was chosen according to a standard dye intensity and based on comparisons with underlying plaque histology, it is to some extent arbitrary. Hence, the percentage of samples defined to be positive for NIRAF is dependent on this cut-off definition. However, a more meaningful cut-off point can only be defined via follow-up studies determining the occurrence of adverse cardiovascular events correlated to various levels of NIRAF.

The current study describes NIRAF as a feature of atherosclerotic plaques that contain intraplaque hemorrhage. Our histological characterization indicates that these plaques are unstable and prone to rupture. However, this does not exclude that some plaques without intraplaque hemorrhage and thus without NIRAF can also cause cardiovascular complications. Ultimately, a definitive association of NIRAF with cardiovascular risk can only be made with certainty, if natural history studies with clinical follow-up are obtained. Such studies are warranted to determine the clinical relevance of NIRAF for the management of atherosclerotic disease and its potential impact on cardiovascular mortality and morbidity.

## Methods

### Human and mouse tissue samples

Human CEA specimens were collected immediately after being taken out at the operating rooms from patients who presented to the Alfred Hospital, Melbourne, Australia with clinical indications for CEA. The project was approved by The Alfred Hospital Ethics Committee (approval number 130/11) and all the participants signed the informed consent form. All animal experiments were approved by the Alfred Medical Research Education Precinct (AMREP) Animal Ethics Committee (approval number E/0998/2010/B).

### Odyssey infrared imaging system

The Odyssey Infrared Imaging System is a closed chamber analyzer that was originally designed for the characterization and quantification of protein bands in gels. It is a bottom up scanning process made possible by two solid state diode lasers with an excitation at 685 nm in the 700 Channel or an excitation at 785 nm in the 800 channel. The detector uses a silicon avalanche photodiode with a resolution of 21–337 µm and adjustable focusing range at 0–4 mm, the scanning speed is between 5 and 40 cm s^−1^. The dye IRDye800CW (LI-COR Biotechnology) was used as a standard for comparison. All figures and fluorescence intensity measurements using the Odyssey Infrared Imaging System were taken using the 800 nm channel unless specified otherwise.

The fluorescence signals from human and murine samples were analyzed using Odyssey software version 3.0.30. For NIRAF analysis, samples were typically prepared fresh. However, paraffin-embedded samples also demonstrated NIRAF. Settings were adjusted to eliminate background fluorescence for image presentation. Absolute MFI values were used for statistical assessment. For human CEA samples, although determined in comparison to IRDye800CWl as a standard dye and to histological characteristics of plaque instability, a relatively arbitrary cut-off fluorescence unit of 20 was used to define the presence ( ≥ 20) or absence (<20) of relevant NIRAF of the samples.

### Raman autofluorescence imaging

Raman autofluorescence imaging of tissue sections was performed on a WITec confocal CRM alpha 300 Raman microscope. The spectrometer was equipped with a Toptica diode laser operating at 785 nm and a back illuminated CCD detector, cooled to −60 °C. The laser was coupled to a microscope via a single mode optical fiber with a diameter of 100 μm and the typical power at the sample was 5 mW using a ×50 objective. The back scattered light was focused onto a multi-mode fiber (100 μm diameter) coupling to a 300 line grating spectrograph. The system was equipped with a long Pass Raman Filter E-Grade and a Razor Edge Dichroic Beamsplitter with high transmission starting at 65 cm^−1^. The integration time for a single spectrum was 0.3 s with a spectral resolution of 3 cm^−1^. To generate autofluorescence images the integrated area between 4000 and 150 cm^−1^ was calculated, which corresponds to integrating the region between 794 and 1144 nm, when taking into account the Stokes shift from the 785 nm excitation line. The grating spectrograph was calibrated using the Raman scattering line produced by a silicon wafer (520.7 cm^−1^). Following excitation at 785 nm, emission fluorescence of the sample between 794 and 1144 nm was detected and presented as shown. Because of the strong fluorescence in this range, no Raman bands were observed and the resulting images represent autofluorescence from the sample.

### Resonance raman spectroscopy

Human CEA samples (with or without autofluorescence) and carotid segments of mice that had undergone TS surgery (generating segments with or without autofluorescence), embedded in “optimal cutting temperature” compound (Sakura Finetechnical, UK) were sectioned at 5 µm (for human samples) and 40 µm (for murine samples), respectively using the Leica CM1950 cryostat (Leica Microsystems Pty. Ltd., Germany). The samples were then mounted on microscope glass slides (Superfrost-Plus, Thermo Scientific, USA). Raman signals were obtained at 14 mW with a 413 nm excitation using the Renishaw InVia confocal micro-Raman system (Renishaw plc, UK) with the Innova Ar/Kr ion laser 514/413 nm as a laser source. The data were analyzed by OPUS 6.0 software (Bruker Optik GmbH).

### Bilirubin extraction from plaque tissue

The whole extraction procedure was performed with minimal exposure to light since bilirubin is light sensitive. The tissue segments were homogenized manually in 1 ml of PBS without Ca^2+^ and Mg^2+^ in Eppendorf tubes. One milliliter of chloroform (Sigma-Aldrich) was added into each tube and vortexed vigorously for 30 s. The tubes were then centrifuged at 1000 × *g* for 10 min. The organic phase was carefully collected and transferred to new tubes for analysis. The tubes were protected from light with aluminum foil.

The Cary 60 UV–vis Spectrophotometer (Agilent technologies, Santa Clara, USA) was used to measure bilirubin concentration in the extracted fluid. The baseline measurements were performed using chloroform first and the absorbance at 476 nm (representing bilirubin concentration) was measured for each sample. A protocol used by a routine laboratory for the quantification of bilirubin in CSF (xanthochromia) was followed^73^.

### Detection of heme, biliverdin and bilirubin by LC–MS/MS

Mouse unstable (segment I, 1–2 mg tissue wet weight) and stable plaques (segment V, 5–9 mg) collected at 1, 4 and 7 weeks post-TS surgery were homogenized in 100 or 200 μl ice-cold PBS (containing 2× protease inhibitor cocktail, Roche Diagnostics), respectively. Homogenization was performed on ice for 3 min using a 0.2 ml Potter-Elvehjem micro tissue grinder (Wheaton). Internal standards (2.25 μl of 15 μM meso-bilirubin, 2.25 μl of 1 μM meso-biliverdin and 2.25 μl of 5 μM n-methyl-protoporphyrin IX) were then added to 50 μl of homogenates followed by 2.25 μl of 0.2 M EDTA. The mixture was mixed for 5 s and then 391 μl of ice-cold methanol containing 0.2 M EDTA added, the mixture vigorously mixed for 30 s and left on ice for 5 min. The extract was then centrifuged at 13,000 × *g* for 10 min at 4 °C and 5 μl of the resulting supernate subjected to LC–MS/MS analysis using an Agilent 1290 binary pump HPLC couple to an Agilent 6490 triple quadrupole mass spectrometer.

The pigments and internal standards were separated on a C-18 column (Zorbax Eclipse Plus C18, RRHD 2.1×50 mm, 1.8 μm) using a gradient of mobile phase A (59.5% water/40% acetonitrile/0.5% acetic acid) and mobile phase B (7.5% water/92% acetonitrile/0.5% acetic acid) at a flow rate of 0.2 ml min^−1^. The gradient was as follows: 0–100% B over 8 min, 100% B for the subsequent 8 min, returning to 0% B over 1 min and then equilibrating at 0% B for 3 min. Following separation, flow was directed into the triple quadrupole mass spectrometer with parameters set as follows: gas temperature = 200 °C; gas flow = 15 l min^−1^; nebulizer pressure = 35 psi; sheath gas heater = 395 °C; sheath gas flow = 11 l min^−1^; capillary voltage = 3500 V and nozzle voltage = 1500 V. Pigments and internal standards were detected by multiple reaction monitoring (MRM) in positive ion mode using the above general mass spectrometry parameters with fragmentor voltage set at 380 V and cell accelerator voltage at 7 V. In each case, the fragment ions generated by collision-induced dissociation of the [M + H]^+^ ions were used for quantification. MRM transitions for the target analytes were: heme (*m*/*z* 616.2→557.2) with collision energy (CE) = 41 V; biliverdin (*m*/*z* 583.3→297.2) with CE = 33, bilirubin (*m*/*z* 585.3→299.2) with CE = 17 V; n-methyl-protoporphyrin IX (*m/z* 577.7→503.3) with CE = 45; meso-biliverdin (*m/z* 587.7→300.3) with CE = 41 and meso-bilirubin (*m/z* 589.7→301.1) with CE = 17. All analytes were quantified against authentic commercial standards obtained from Frontier Scientific and data was normalized to tissue wet weight.

### FLECT

ApoE^−/−^ mice were placed on a western diet for 13 weeks (6 weeks before and 7 weeks after TS surgery), as described previously. On the day of imaging, mice were anesthetized, shaved and imaged in the FLECT scanner (Bioscan, Washington DC) to obtain 3-dimensional acquisition using a 730 nm excitation laser, 803 nm filter with 29 slices at 1 mm spacing and 29 source angles per slice. The reconstruction was performed using 1 mm^3^ grid with attenuation correction at 1000 iterations to generate the reconstructed three-dimensional image of each scan. Following the FLECT scan, the Computed Tomography (CT) scan was performed for each animal using settings of 45 kV, 1500 ms & 360 projections to generate the anatomical image, which was overlayed onto its respective FLECT image.

### Heme and associated products

Tetrapyrole-containing compounds used in this manuscript were all purchased from Sigma-Aldrich, USA): Bilirubin (cat. no.: B4126), Hemoglobin (cat. no.: H7379), Ferrous stabilized hemoglobin (cat. no.: H0267), Biliverdin hydrochloride (cat. no.: 30891), Protoporphyrin IX (cat. no.: P8293), Protoporphyrin IX cobalt chloride (cat. no.: C1900), and Protoporphyrin IX zinc(II) (cat. no.: 282820).

### Fluorescence microscopy

Fluorescence images were recorded on a DeltaVision Widefield microscope (Applied Precision) using a ×60 1.42 NA oil immersion objective, a Cy5 filter set (excitation: 650/13, emission: 684/24) for Alexa 647 fluorescence and a Cy7 filter set (excitation: 740/13, emission: 809/81) for NIR autofluorescence. The system is equipped with a Photometrics HQ camera. Images were recorded with 4 × 4 pixel binning and critical illumination to be able to detect the NIR autofluorescence signal. Bright field images were recorded along with fluorescence images. Image processing was performed using the open-source image analysis software Fiji.

### Old and new blood preparation

Fresh whole blood was obtained from mice via cardiac puncture using 1 ml Terumo syringes with 25 G needles. Heparin was used as anticoagulant at a total volume of 100 μl (final concentration 20 IU ml^−1^). For old blood with and without anticoagulant, blood remained 45 min at room temperature. Syringes with blood were scanned using the Odyssey Infrared Imaging System at 21 μm resolution.

### Immunohistochemistry

The 5 μm cryosections of human CEA samples were thawed at room temperature for 30 min and then fixed in acetone at −20 °C for 20 min. Samples were sequentially fixed again in 10% neutral buffered formalin at room temperature for 2 min and absolute ethanol for 10 min to reduce background. Subsequently, all slides were treated with 3% hydrogen peroxide for 30 min to block endogenous peroxidase activity. Sections were incubated with normal rabbit serum for 30 min, followed by avidin and biotin blocking according to manufacturer’s requirement (Vector Laboratories, CA, USA). After the blocking steps, the sections were treated with primary antibodies for 1 h. Anti-bilirubin (clone 24G7, Shino-test, Kanagawa, Japan, dilution 1:100), anti-hemoglobin subunit alpha (cat no: ab102758, Abcam, USA, dilution 1:100) and anti-glycophorin A (human samples: cat no: ab129024, Abcam, USA, dilution 1:100; mouse samples: cat. no.: bs8042, Bioworld Technology, USA, dilution 1:100) were used as primary antibodies to detect intraplaque hemorrhage. Sections were subsequently incubated with corresponding biotinylated secondary antibodies for 30 min, followed by incubation with the avidin-biotin-peroxidase system (Vectastain ABC kit, Vector Laboratories). The reaction products were stained with a DAB substrate kit for peroxidase (Vector Laboratories) to generate a brown reaction product. Then sections were counterstained with hematoxylin. Negative controls were done identical to the above protocol, except for incubation with PBS instead of primary antibodies.

### Iron staining based on prussian blue reaction

Iron stain kit from Sigma-Aldrich, Australia was used for the procedure. The 5 μm paraffin sections of human CEA samples were deparaffinized and then hydrated with deionised water. The samples were then treated with Working Iron Stain Solution prepared by mixing equal volumes of potassium ferrocyanide solution and hydrochloric acid solution. The slides were then rinsed with deionized water and stained with Working Pararosaniline solution (prepared by 1 ml of Pararosaniline solution to 50 ml of water) for 5 min. The slides were rinsed again with deionized water and hydrated with alcohol and xylene before examining for Prussian blue reaction (bright blue staining of iron).

### IVIS fluorescence scanning of hemoglobin and bilirubin embedded in agarose

Hemoglobin and bilirubin (50 μg each) were discretely embedded in an agarose phantom (10% w/v) before imaging in the IVIS Lumina Series II Imaging System (Caliper Life Sciences, MA, USA). Fluorescence images were obtained by a charge-coupled device (CCD) camera using the XFO-12 fluorescence equipment with appropriate filter combinations (excitation filters for 605 nm, 640 nm, 675 nm, 710 nm, 745 nm and emission filter for 810–875 nm). Reflected light photographic pictures were taken during illumination with white light-emitting diode. Both fluorescence and photographic images were overlayed, processed and analyzed using Living Image 4.4 software.

### Tandem stenosis surgery

At 12 weeks of age, 6 weeks after commencement of HFD, ApoE−/− mice (C57BL/6J background) were anaesthetized by ketamine (80 mg kg^−1^) and xylazine (20 mg kg^−1^) mixture through intraperitoneal injection. An incision was made in the neck and the right common carotid artery was dissected from circumferential connective tissues. A TS with 150 μm outer diameters was introduced with the distal point 1 mm from the carotid artery bifurcation and the proximal point 3 mm from the distal stenosis. The stenosis diameter was obtained by placing a 6-0 blue braided polyester fiber suture (TI.CRON 0.7 Metric) around the carotid artery together with a 150 μm needle (Ethicon 8-0, Virgin silk blue, W1782) that was tied to it and later removed. Animals were euthanized at 7 weeks after surgery.

### Statistical analysis

GraphPad Prism software (GraphPad Software, Inc., La Jolla, CA, USA) was used for all statistical analyses. Unpaired Student’s *t*-test or Mann–Whitney *U* test was used for the comparison of parameters between two groups in Figs. 2b and 4b. The Kruskal–Wallis test followed by Dunn’s multiple comparisons test were used for comparison of parameters among three groups in Figs. 4c, 7b and c. Parameters were shown quantitatively as mean ± SEM. A *p* value of <0.05 was considered statistically significant.

### Data availability

The data that support the findings of this study are available from the corresponding author upon reasonable request.

## Electronic supplementary material

Supplementary Information

Supplementary Movie 1

Supplementary Movie 2
