# Supplementary material for: Near-infrared autofluorescence induced by intraplaque hemorrhage and heme degradation as marker for high-risk atherosclerotic plaques
Source: Nat Commun. 2017 Jul 13;8:75. doi: 10.1038/s41467-017-00138-x (PMC5509677; doi:10.1038/s41467-017-00138-x)
Supplement: Supplementary file 1 — Supplementary Information [file 41467_2017_138_MOESM1_ESM.pdf]

File name: Supplementary Information
Description: Supplementary figures, supplementary table and supplementary references.

File name: Supplementary Movie 1
Description: 3D reconstruction movie of a TS mouse with a fluorescent plaque imaged via FLECT/CT as described in Figure 8A.

File name: Supplementary Movie 2
Description: 3D reconstruction movie of a TS mouse with non-fluorescent plaque imaged via FLECT/CT as described in Figure 8B.

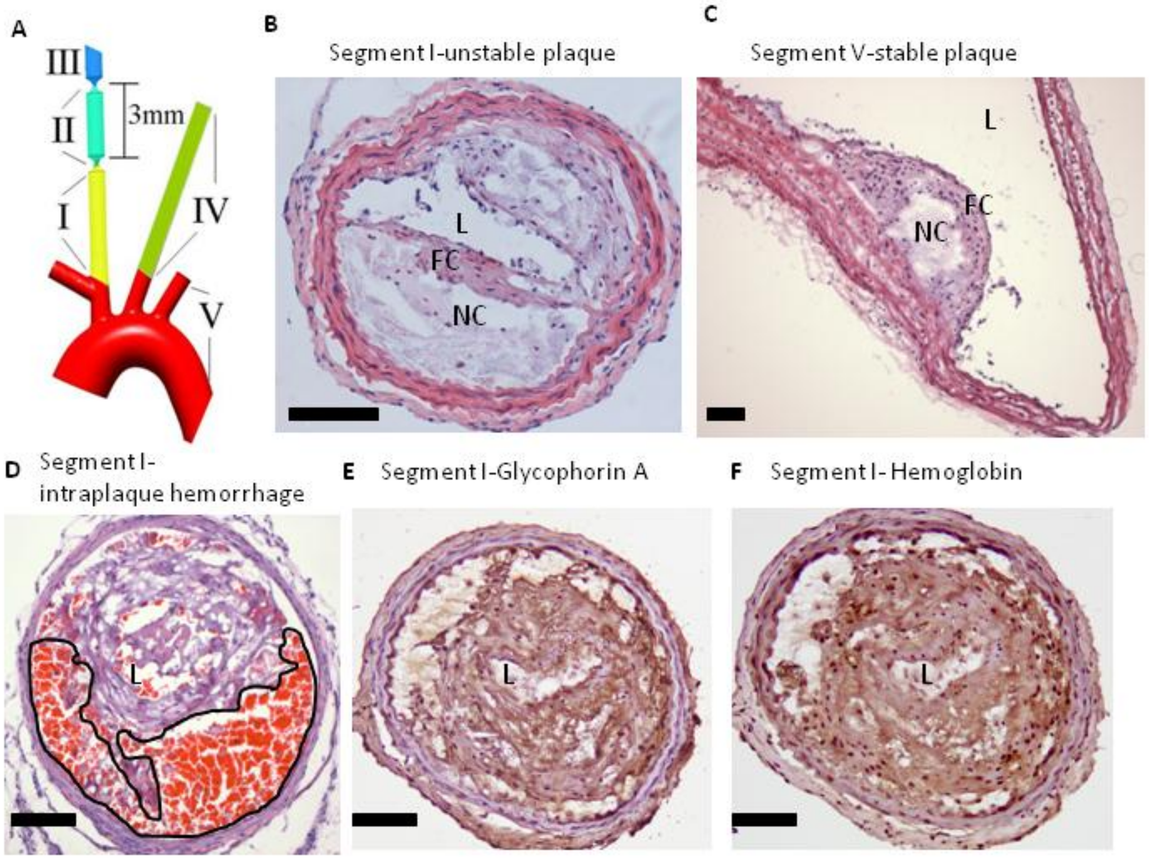

Supplementary Figure 1: Tandem Stenosis (TS) mouse model and examples of histologically defined unstable plaques (Segment I) and stable plaques (Segment V). (**A**) Schematic drawing of segment I to V of the TS model with the two ligations 3 mm apart in the right carotid artery. (**B**) Segment I (the carotid artery segment proximal to the two ligations) represents the area where spontaneous intraplaque hemorrhage and thin cap fibroatheromas occured. (**C**) Segment V represents the aortic arch including the proximal portions of brachiocephalic, left carotid and left subclavian arteries, where the atheromatous lesion phenotypes typically included thick caps with small necrotic cores and no intraplaque hemorrhages. (**D**) Histological section of segment I using hematoxylin and eosin staining. Intraplaque hemorrhage in segment I was seen in around 50% of mice after TS surgery. (**E**) Immunohistochemical staining for glycophorin A demonstrated the presence of red blood cells in segment I. (**F**) Segment I also showed a positive immunohistochemical staining for hemoglobin. Experiments were performed 35 times and representative examples are shown. Scale bars indicate 100 µm.

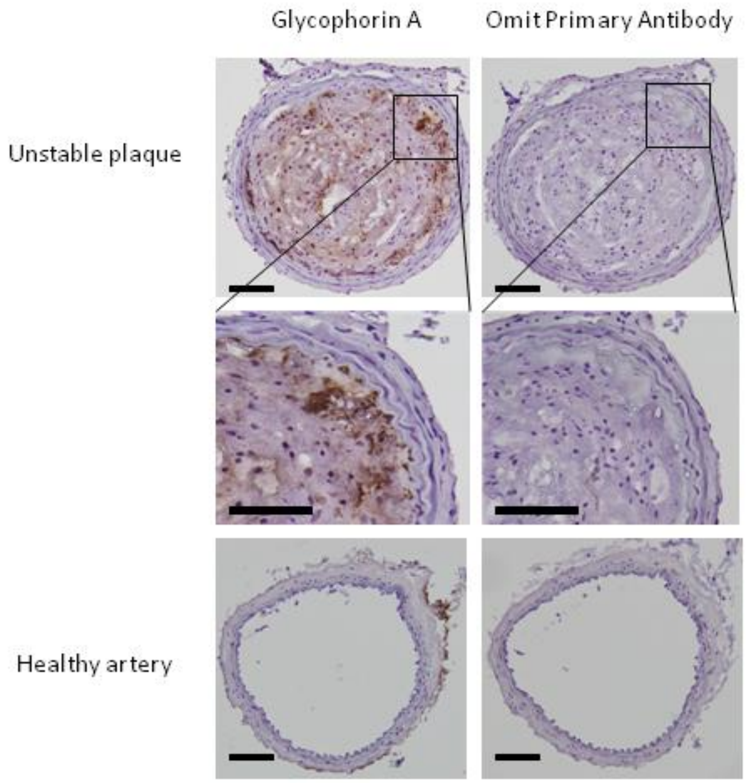

Supplementary Figure 2: Immunohistochemistry of glycophorin A in unstable athero-sclerotic plaques. Glycophorin A staining shows a strong signal in the hemorrhagic plaque region (section adjacent to segment-c in Figure 2D) in comparison to the negative control (no primary antibody). Experiments were performed 3 times and one representative example is shown. Scale bars indicate 100 µm.

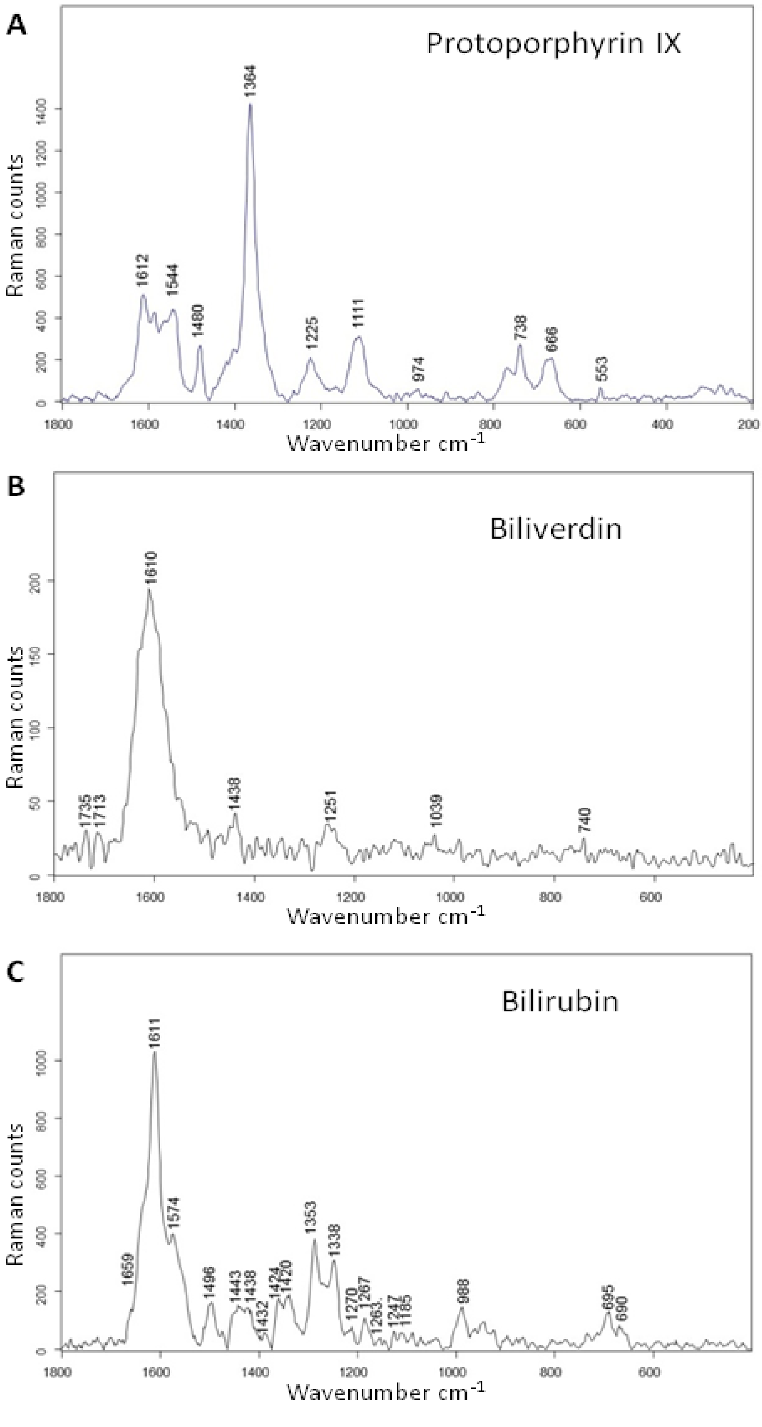

Supplementary Figure 3: Raman spectra of protoprophyrin IX, biliverdin and bilirubin. Raman spectra obtained from different heme-related chemicals showed similar peaks (backbone of heme compounds) at 1364 - 1360 cm^{-1} (wave numbers) region. The spectra of biliverdin and bilirubin differ markedly from the hemoglobin and protoporphyrin IX spectra because the porphyrin structure no longer exists in biliverdin and bilirubin. (**A**): Raman signals from protoporphyrin IX, which is the precursor of heme. (**B**): Raman signals from biliverdin (the breakdown product of heme via by the enzyme heme oxygenase 1). (**C**): Raman signals from bilirubin (the breakdown product of biliverdin mediated by biliverdin reductase).

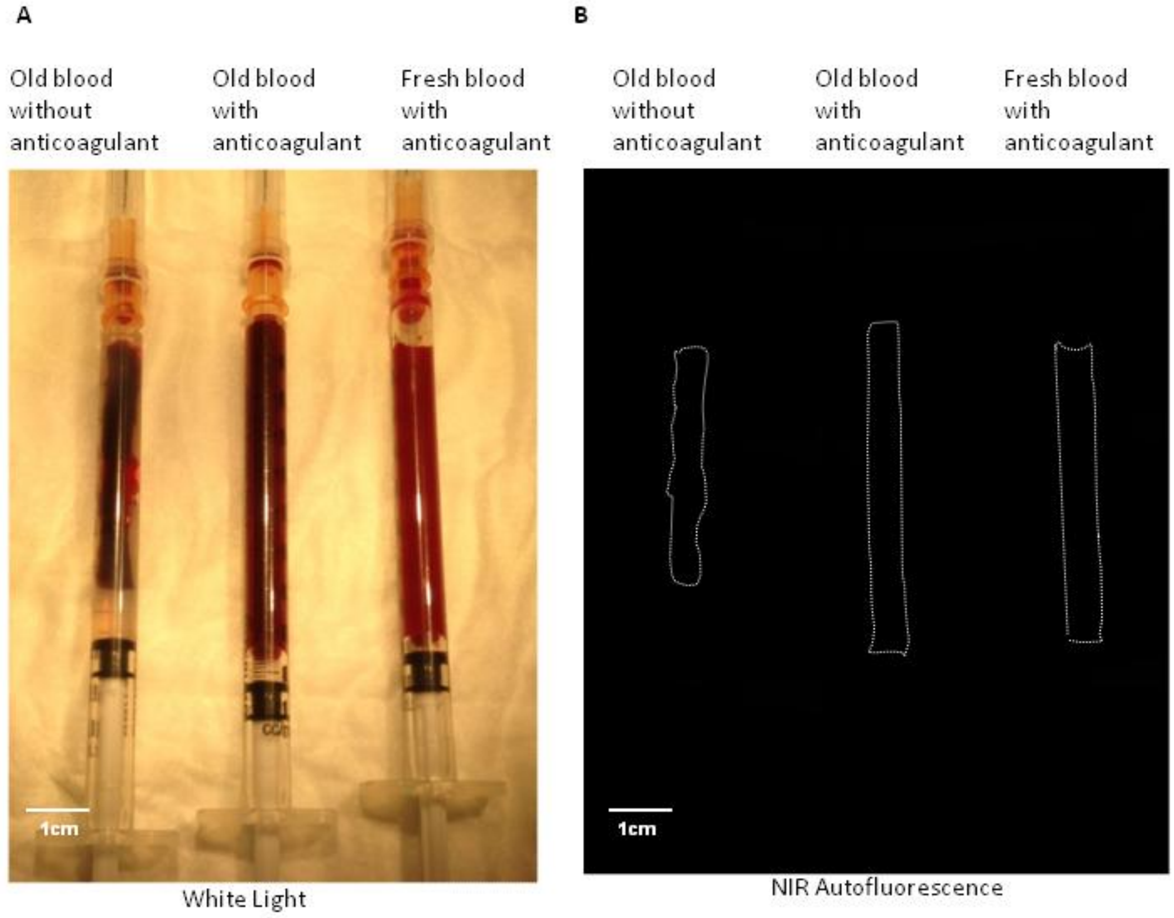

Supplementary Figure 4: Old blood with or without anticoagulant and fresh blood with anticoagulant did not show any NIRAF. (**A**): White light pictures of old blood with or without anticoagulant and fresh blood with anticoagulant. (**B**): Odyssey Infrared Imaging System images of old blood with or without anticoagulant and fresh blood with anticoagulant demonstrated no NIR autofluorescence. Dash box indicated the position of blood samples in NIR images. Experiments were performed 3 times and one representative example is shown. Scale bars indicate 1 cm.

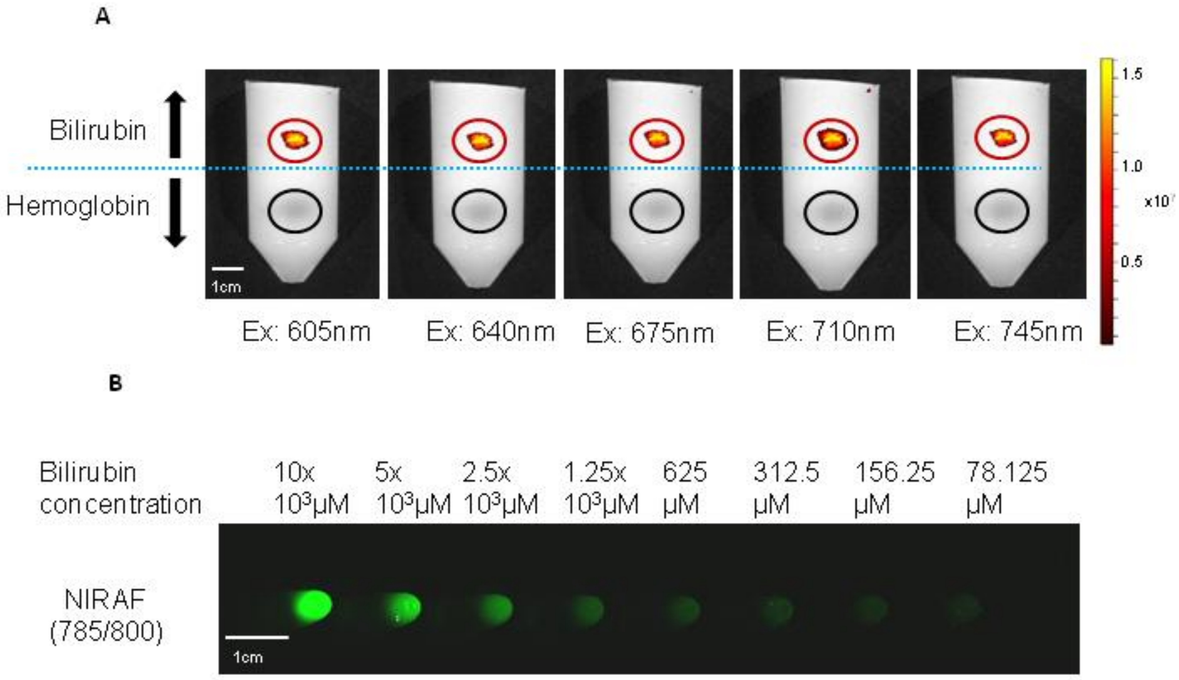

Supplementary Figure 5: Bilirubin and hemoglobin fluorescence scanning at various excitation wavelengths. (**A**): Bilirubin (50 µg; the top red circle) and hemoglobin (50 µg; the bottom black circle) were embedded in an agarose phantom. The phantom was then scanned on the IVIS imager using different excitation wavelengths (605, 640, 675, 710 and 745 nm) to detect NIR signals above 810 nm. The signal intensity (measuring Radiant Efficiency) is shown as a color bar on the right. Bilirubin but not hemoglobin showed strong NIR signals at all excitation wavelengths from 605 to 745 nm. (**B**): Bilirubin dissolved in chloroform at various concentrations (serial dilution) as a positive NIRAF control. NIRAF was detected both on the IVIS imager (Ex 605 to 745 nm) and the Odyssey Infrared Imaging System (shown as example) at Ex 785 nm, Em > 800 nm. Experiments were performed 3 times and one representative example is shown. Scale bars indicate 1 cm.

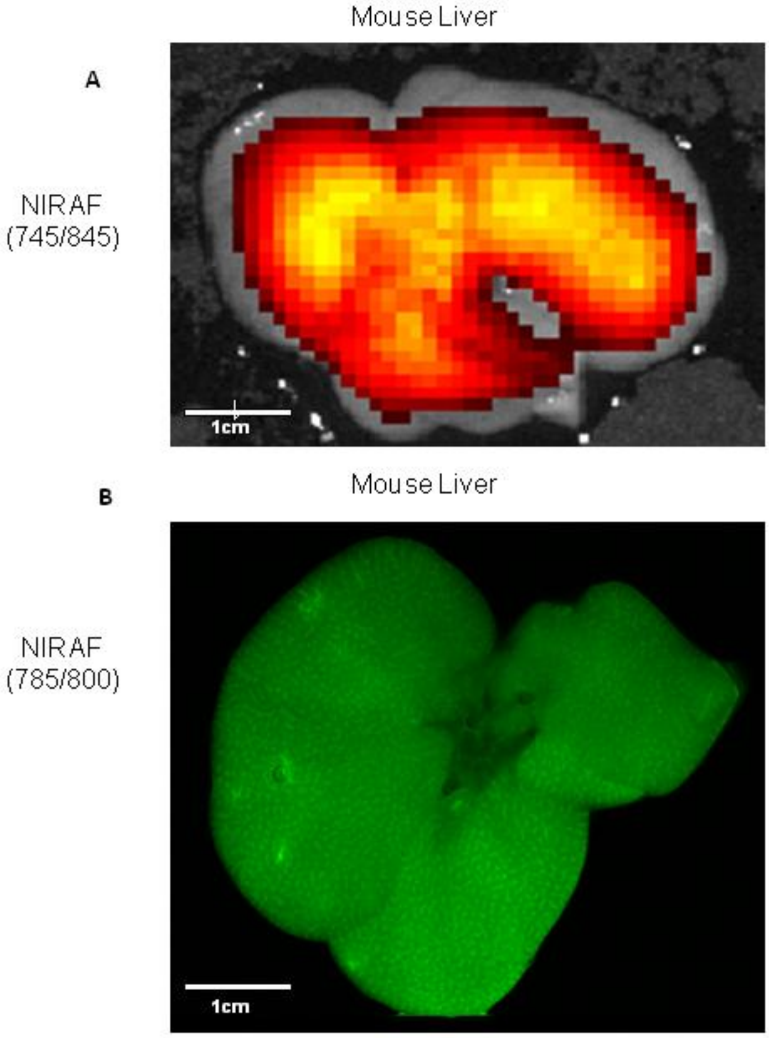

Supplementary Figure 6: Representative images of a mouse liver exhibiting strong NIRAF consistent with the high abundance of bilirubin in the liver. (**A**) IVIS images were obtained at Ex 745 nm, Em 845 nm for detection of liver NIRAF. Similar fluorescence pictures were obtained between 405 and 710 (see supplementary figure 4A) (**B**) Odyssey NIRAF images were also obtained at Ex 785 nm, > Em 800 nm, intensity level 5, resolution at 21 µm. Experiments were performed 3 times and one representative example is shown. Scale bars indicate 1 cm.

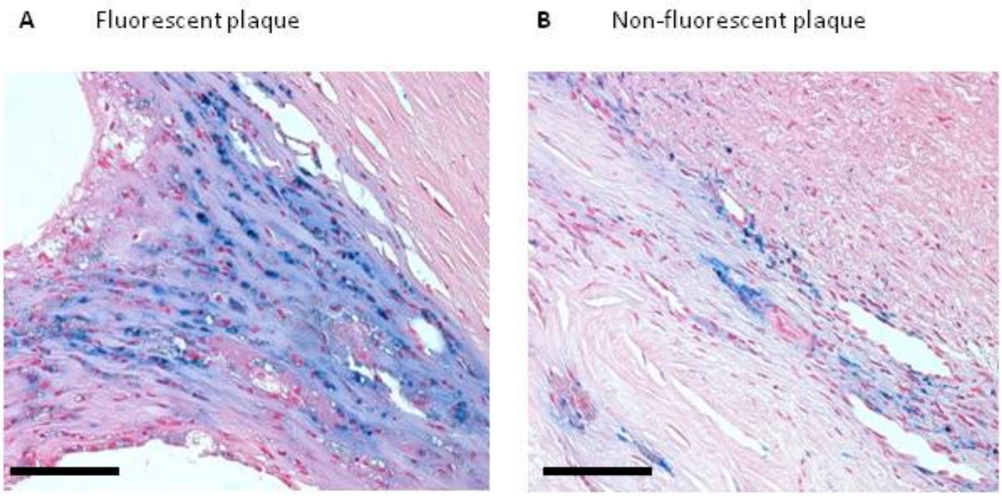

Supplementary Figure 7: Prussian blue staining of human CEA samples with or without NIRAF. (**A**): CEA sample with NIRAF showing positive Prussian blue reaction, which indicates the presence of hemosiderin granules from intraplaque hemorrhage. (**B**): CEA sample without NIRAF shows only minor Prussian blue reaction. Experiments were performed 6 times and one representative example is shown. Scale bars indicate 100 µm.

Supplementary References:

1. S. Hu, K.M. Smith, T.G. Spiro. Assignment of protoheme resonance Raman spectrum by heme labeling in myoglobin. *J. Am. Chem. Soc.* **118,** 12638-12646 (1996).

2. Wood, B.R., Tait, B. & McNaughton, D. Micro-Raman characterisation of the R to T state transition of haemoglobin within a single living erythrocyte. *Biochim. Biophys. Acta* **1539**, 58-70 (2001).

Supplementary Table 1:

Observed bands, assignments, local coordinates and symmetry terms for heme moieties observed at 632.8 nm.

oxyRBC	deoxyRBC	Haemin	Haematin	HbA_0	metMb	Mode [1]	Local coordinate [1][a]	Symmetry [1][b]
1636 (dp)	absent	absent	absent	1637	1636[c]	X_{10}	$X(C_{\kappa}C_{m})_{asym}$	B_{1g}
1617	1618 sh	1626	1626	1618	1621	$X(C_a = C_b)$	$X(C_a = C_b)$	
1606 (ap)	1606	absent	absent	1606	1606	X_{19}	$X(C_{\kappa}C_{m})_{asym}$	A_{2g}
1582 Sh	1580	1584 sh	1584	1582	1570[c]	X_{37}	$X(C_{\kappa}C_{m})_{asym}$	E_u
1562 (p)	absent	1568	1568	1562	1558[c]	X_2	$X(C_{L}C_{L})$	A_{1g}
1547 (dp)	1544	1548	1549	1544	1548[c]	X_{11}	$X(C_{L}C_{L})$	B_{1g}
1430 (dp)	1428	1429	1430	1430	1426	X_{28}	$X(C_{\kappa}C_{m})_{sym}$	B_{2g}
1395 (ap)	1393	1398	1397	1396	1391	X_{20}	X(pyr quater-ring)	A_{2g}
1367 (p)	1367	1371	1374	1365	1362	X_4	X(pyr half-ring)$_{sym}$	A_{1g}
1338	1334	1339	1339	1341	1340	X_{41}	X(pyr half-ring)$_{sym}$	E_u
1306 (ap)	1306	1306	1307	1306	1311	X_{21}	$N_{asym}(C_m H)$	A_{2g}
1245	absent	absent	absent	1245	1246	X_{13}	$N(C_m H)$	B_{1g}
1224 (dp)	1224[c]	1234	1234	1225	1224[c]	X_{13} or X_{42}	$N(C_m H)$	B_{1g} or E_u
1210[c] (dp)	1215	1219	1219	1209[c]	1210[c]	X_5 X	N(C H)	B
1169 (dp)	1169	1167	1169	1172	1170	X_{30}	X(pyr half-ring)$_{asym}$	B_{2g}
1122 (ap)	1117	1122	1121	1122	1125	X_{22}		
1091 (ap)	1088	1087	1091	1078	1084	X_{23}	$X(C_{L}C_{1})_{asym}$	A_{2g}
995 (dp)	997	1001	1002	997	997	X_{45}	$X(C_{L}C_{1})_{asym}$	E u
976 (dp)	976	972	973	975	974	X_{46}	N(pyr deform)$_{asym}$ and/or $Q(=C_b H_2)_{sym}$	E_u
823 (dp)	827	825	825	828	827	Q_{10}	$Q(C_m H)$	B_{1u}
789 (p)	791	792	797	793	792	X_6	X(pyr breathing)	A_{1g}
752 (dp)	755	754	753	753	754	X_{15}	X(pyr breathing)	B_{1g}
667 (p)	676	absent	absent	664	666	X_7	N(pyr deform)$_{sym}$	A_{1g}

RBC, red blood cell. p, polarized; dp, depolarised; ap, anomalously polarised; X, stretch; N, in-plane deformation; Q, out-of-plane deformation; sym, symmetric; asym, asymmetric; pyr, pyrrole; deform, deformation; w, weak band relative to others.

[a]Subscripts refer to haem labelling scheme adopted by Hu *et al.* [1].

[b]Based on calculated depolarization ratios for oxyRBC and by comparison with Hu *et al.* [1].

[c]Only observed after calculating second derivative.

See also Wood *et al.* [2].
