# Supplementary figures and images for: Near-infrared autofluorescence induced by intraplaque hemorrhage and heme degradation as marker for high-risk atherosclerotic plaques
Source: Nat Commun. 2017 Jul 13;8:75. doi: 10.1038/s41467-017-00138-x (PMC5509677; doi:10.1038/s41467-017-00138-x)

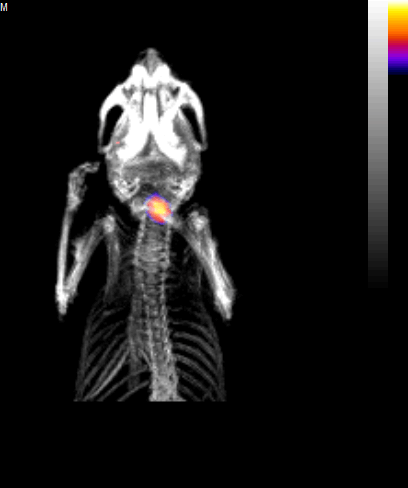

Supplement: Supplementary file 2 — Supplementary Movie 1 [file 41467_2017_138_MOESM2_ESM.gif]

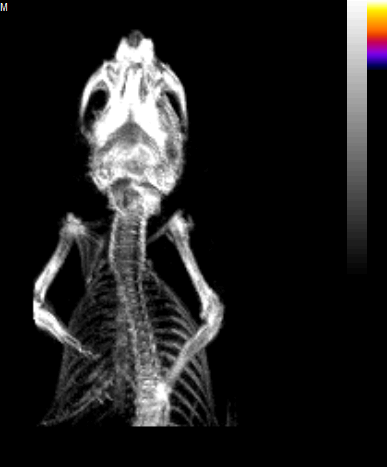

Supplement: Supplementary file 3 — Supplementary Movie 2 [file 41467_2017_138_MOESM3_ESM.gif]
